# Prevalence of cough and associated symptoms among pilgrims in large mass gathering event 2024: a cross-sectional study

**DOI:** 10.1016/j.ijregi.2025.100733

**Published:** 2025-08-15

**Authors:** Anas Khan, Fahad Alamri, Reem Hasan, Mariyyah Alburayh, Ghadah Alsaleh, Areej Alshamrani, Hala Aljishi, Jaffar Al-Tawfiq

**Affiliations:** 1 Department of Emergency Medicine, College of Medicine, King Saud University, Saudi Arabia; 2 Global Centre of Mass Gatherings Medicine, Family Medicine, Primary Health Centre, Ministry of Health, Riyadh, Saudi Arabia; 3 Global Centre for Mass Gatherings Medicine, Ministry of Health, Riyadh, Saudi Arabia; 4 Specialty Internal Medicine and Quality Department, Johns Hopkins Aramco Healthcare, Dhahran, Saudi Arabia; 5 Indiana University School of Medicine, Indianapolis, USA; 6 Division of Infectious Disease, Department of Medicine, Johns Hopkins University School of Medicine, Baltimore, USA

**Keywords:** Hajj, Cough, Respiratory symptoms, Mass gatherings, Infectious diseases, Public Health, Chronic diseases

## Abstract

**Objectives:**

Large mass gathering events significantly increase the risk of infectious disease transmission, particularly respiratory infections, due to unavoidable overcrowding and exposure to airborne pathogens. Therefore, this study aims to assess the prevalence of cough, its duration, and associated symptoms during the religious mass gathering event among pilgrims in the 2024 Hajj season.

**Methods:**

This cross-sectional study was conducted during Hajj in Makkah, Saudi Arabia, in 2024. A face-to-face random interview utilizing a structured questionnaire was employed to collect data from 2,913 pilgrims, who were randomly selected as participants and were at least 18 years old. Baseline demographic data and clinical characteristics were compiled using descriptive statistics. Continuous variables were presented as means and standard deviations, while categorical data were illustrated as counts and percentages.

**Results:**

Among 2913 Hajj pilgrims, the average age was 53.9 ± 11.8 years, and 1,173 (40.4%) reported cough symptoms. The highest prevalence was in the 50–64 age group (60.7%). Chronic diseases were significantly more common in patients with cough (53.3%). Diabetes (357 cases) and hypertension (330 cases) were the most common conditions. Of the 1,173 participants with cough, 10.3% reported no associated symptoms, while sore throat (30.8%) was the most common. Logistic regression confirmed chronic disease, nationality, and age as significant predictors of cough.

**Conclusions:**

A significant number of cough symptoms were reported, with the highest incidence in older adults. Additionally, notable associations were identified between cough and pre-existing health conditions, particularly diabetes mellitus, hypertension, chronic heart disease, and asthma.. Future research should investigate the long-term effects of cough and its related symptoms or use of medications in mass gatherings.

## Highlights

• The study thoroughly examined the prevalence of cough and related symptoms among pilgrims during the 2024 Hajj season’s large religious mass gatherings.
• The highest cough prevalence is among older age group.
• There is a significant association between experiencing cough with pre-existing health condition.
• The logistic regression analysis revealed that chronic diseases, age, and nationality were significant predictors of cough among the participants.
• The results indicate that respiratory health among pilgrims during the Hajj season is significantly influenced by age and the presence of chronic diseases.

## Introduction

Mass gatherings (MGs) encompass religious events such as Hajj, Christian festivals, and Kumbh Mela, as well as sports events like the Olympics and the soccer World Cup. MGs are associated with increased risks of respiratory infections. For example, upper respiratory tract infections (URTIs) have a prevalence ranging from 50% to 93% among attendees [[1], [2], [3]]. These gatherings created environments that facilitate the acquisition, dissemination, and transmission of pathogenic microorganisms, leading to a higher incidence of infections.

Of the MGs, Hajj poses unique challenges that heighten the risk of respiratory infections.

The annual Hajj attracts millions of pilgrims from diverse backgrounds, creating conditions that facilitate rapid pathogen spread. Pilgrims may encounter various pathogens, including influenza, respiratory syncytial virus (RSV), and coronaviruses, which can lead to URTIs characterized by coughing, sneezing, and nasal congestion [4,5].

A recent nested case-control study involving Iranian pilgrims found that the time spent in holy places and direct contact with ill pilgrims in shared accommodations did not significantly affect the incidence of respiratory infections [6]. This indicates that factors beyond mere exposure may influence the risk of respiratory illness. In contrast, a study of US pilgrims reported adherence to social distancing and contact avoidance measures as 34.4% and 24.2%, respectively, and these practices were linked to a significant reduction in respiratory symptoms during the Hajj [3]. This underscores that direct contact among pilgrims contributes to respiratory infections. Overall, these studies suggest that the spread of respiratory diseases among pilgrims can be attributed to both direct contact and other influencing factors.

Common symptoms reported by attendees include cough, runny nose, sore throat, and fever, which were particularly prevalent during the Hajj [7]. Additionally, cough is a primary reason individuals seek medical attention from healthcare providers during such events, as it significantly affects the quality of life [8]. This widespread occurrence can be attributed to a combination of environmental, social, and physiological factors at MGs [9,10].

During Hajj from 2012 to 2014, the prevalence of cough among pilgrims was 80.9%, with sore throat (91.0%), rhinitis (78.7%), and hoarseness (63.0%) [4]. In subsequent years, cough prevalence remained significant at 73.6% in 2018 and 42.8% in 2021 [9,11]. This continuity indicates that respiratory illnesses persist during Hajj, irrespective of individual preventive behaviors. Additionally, studies have indicated that females are significantly more likely to experience coughing than males, reporting rates of 85.3% compared to 73.6%, potentially influenced by hormonal differences, immune response variations, and behavioral factors [12]. Furthermore, it was found that cough was the main complaint among individuals over 55 years of age, with an attack rate of 51% [13].

These findings illustrate that both environmental conditions and demographics play critical roles in shaping the respiratory health outcomes of pilgrims. This has led to the phenomenon informally referred to as the “Hajj cough,” which is characterized by persistent, dry coughing that can last several days or even weeks [14,15]. While often self-limiting, the cough can become severe, leading to complications such as pneumonia or exacerbation of chronic conditions. For pilgrims with underlying health issues, such as asthma or cardiovascular disease, the impact of the cough is particularly significant, increasing the risk of hospitalization and other complications [9,10,12].

Other environmental factors during Hajj, such as high temperatures, humidity, crowded spaces, and sudden temperature changes, significantly impact the transmission of respiratory viruses and increase coughing among pilgrims. Hot, dry air is an irritant/pollutant to the respiratory system, while high temperatures can help viruses survive longer in the air, promoting transmission [16]. Humidity may cause respiratory irritation, leading to more coughing, especially in individuals with respiratory conditions. Crowded areas with insufficient ventilation increase the concentration of viral particles, facilitating the spread of infections [17].

Given the continued high incidence of cough during the Hajj, it is essential to take preventive measures to reduce the risk of respiratory infections. Further investigation is needed to determine the specific environmental conditions that exacerbate respiratory symptoms, as well as the demographic variables that may influence susceptibility. Factors such as air quality, temperature fluctuations, and crowd density require closer examination to understand their roles in the transmission of respiratory pathogens. Additionally, exploring the pilgrims’ pre-existing health conditions, vaccination status, and adherence to preventive measures will offer a more comprehensive view of the situation.

This study aims to assess the prevalence, etiology, and severity of cough among Hajj pilgrims during the 2024 Hajj season. By identifying the factors that contribute to the high rates of coughing and respiratory illness, this research will provide better health strategies and interventions to protect pilgrims from respiratory infections.

## Methods

### Study design

This cross-sectional study was done from 25 June to 5 July 2024 in the departure area of King Abdulaziz International Airport, Jeddah, Saudi Arabia, after pilgrims had completed the Hajj rituals of the 1445 H. Questionnaires were randomly distributed across available flight schedules to pilgrims. We collected data on cough symptoms and demographic characteristics, including nationalities, age groups, and gender. All individuals were informed about their participation in the study, and verbal consent was obtained. Throughout the data collection, handling, and storage processes, strict adherence to the ethical standards outlined in the Declaration of Helsinki was maintained to ensure participant confidentiality. The study protocol was approved by the Central Institutional Review Board, Ministry of Health in Riyadh, Saudi Arabia (IRB log NO:24-59 M).

### Inclusion and exclusion criteria

The study included pilgrims aged 18 years or older. We excluded pilgrims who declined to provide informed consent or who failed to supply the requested information.

### Sample size calculation

We utilized the Raosoft® software to calculate the required sample size, aiming for a 95% confidence level, a response rate of 50%, and a margin of error of 2%. Based on these parameters, the estimated sample size was 2,144 participants. The data collection resulted in a total of 4,470 pilgrims. However, after applying specific filtering criteria, the final sample size was adjusted to 2,913 participants, still within the estimated range with a margin of error of 1.78%, while maintaining the 95% confidence level.

### Data collection

Data collection was conducted through an electronic platform and face-to-face random interviews utilizing a structured questionnaire. The questionnaire was available in both Arabic and English. It included demographic data such as age, sex, nationality, and education level; questions about medical history related to chronic disease; and cough-related characteristics, such as duration and associated symptoms. The validity of this questionnaire was initially checked by a public health specialist and a family physician, who confirmed that the instrument met the necessary standards for research. Additionally, a reliability analysis using Cronbach's alpha test on a randomly selected sample of 200 pilgrims during the Hajj 2024 project demonstrated a moderate level of internal consistency [18], with an overall Cronbach’s alpha of 0.6.

### Statistical analysis

Descriptive statistics were employed to summarize the initial demographic and clinical characteristics. Categorical data were displayed as counts and percentages, while continuous data were shown as means with standard deviations. For continuous variables, the *t*-test was applied, and for categorical variables, the chi-squared test was utilized. To determine factors related to the prevalence, etiology, and severity of cough, both simple and multiple logistic regression analyses were conducted, calculating odds ratios, adjusted odds ratios (aOR), and 95% CI. A significance level of *P* < 0.05 was established. The statistical analyses were carried out using MATLAB (R2023b version 23.2).

## Results

### Participant characteristics

The study included 2,913 pilgrims, with an average age of 53.9 ± 11.8 years. Table 1 outlines the demographic characteristics of Hajj pilgrims included in the study. Among these participants, 1,176 (40.4%) reported cough symptoms. The majority of participants were in the age groups of 50–64 years (57.3%) and 30–49 years (32.8%). Within these age groups, the prevalence of cough symptoms was 60.7% and 27.9%, respectively. The male-to-female ratio among participants with cough symptoms was approximately 1:1, consistent with the overall study population. Regarding educational background, a significant portion of participants did not disclose their educational attainment, with 55.3% overall, and 33.7% of those with cough symptoms, not providing this information. Among those with cough symptoms, 18.9% had obtained university degrees and completed intermediate or secondary education. In contrast, the percentages for the entire participant group were 13.9% and 12.7%, respectively. The prevalence of illiterate participants was relatively low, recorded at 6.79% among all participants and 10.21% among those with cough symptoms.

**Table 1:** Demographic characteristics of Hajj pilgrims participating in the study.

Characteristic	Reported cough symptom		Total	
	N	%	N	%
**Total**	1176	40.4 %	2913	100%
**Age group**				
≤29	10	0.90	47	1.63
30-49	328	27.9	956	32.8
50-64	555	60.7	1257	57.3
≥65	283	10.5	653	8.27
**Sex**				
Female	574	48.8	1400	48.1
Male	602	51.2	1513	51.9
**Education level**				
Intermediate or Secondary	222	18.9	371	12.7
University	222	18.9	404	13.9
Read & write	216	18.29	329	11.31
Illiterate	120	10.21	198	6.79
preferred not to say	396	33.7	1611	55.3
**Nationality by World Health Organization Region**				
African Region	306	26.0	803	27.6
South-East Asia Region	182	15.47	708	24.3
European Region	104	8.84	311	10.87
Eastern Mediterranean Region	490	41.7	956	32.8
Western Pacific Region	94	7.99	135	4.63
				
**Chronic disease**	626	53.3	990	33.9

Geographically, the Eastern Mediterranean Region had the highest participation, comprising 32.8% of the total participants and 41.7% of those reporting cough symptoms. Smaller participation rates were observed from the Western Pacific and European Regions, with 8.84% and 7.99% reporting cough symptoms, respectively, compared to participation rates were 10.87% and 4.63%. Among all participants, 33.9% reported having a chronic disease, while 66.1% did not. The prevalence of chronic diseases was higher among those experiencing cough symptoms, at 53.3%, compared to those without chronic conditions but who had a cough.

### Comorbidities

**Figure 1:**
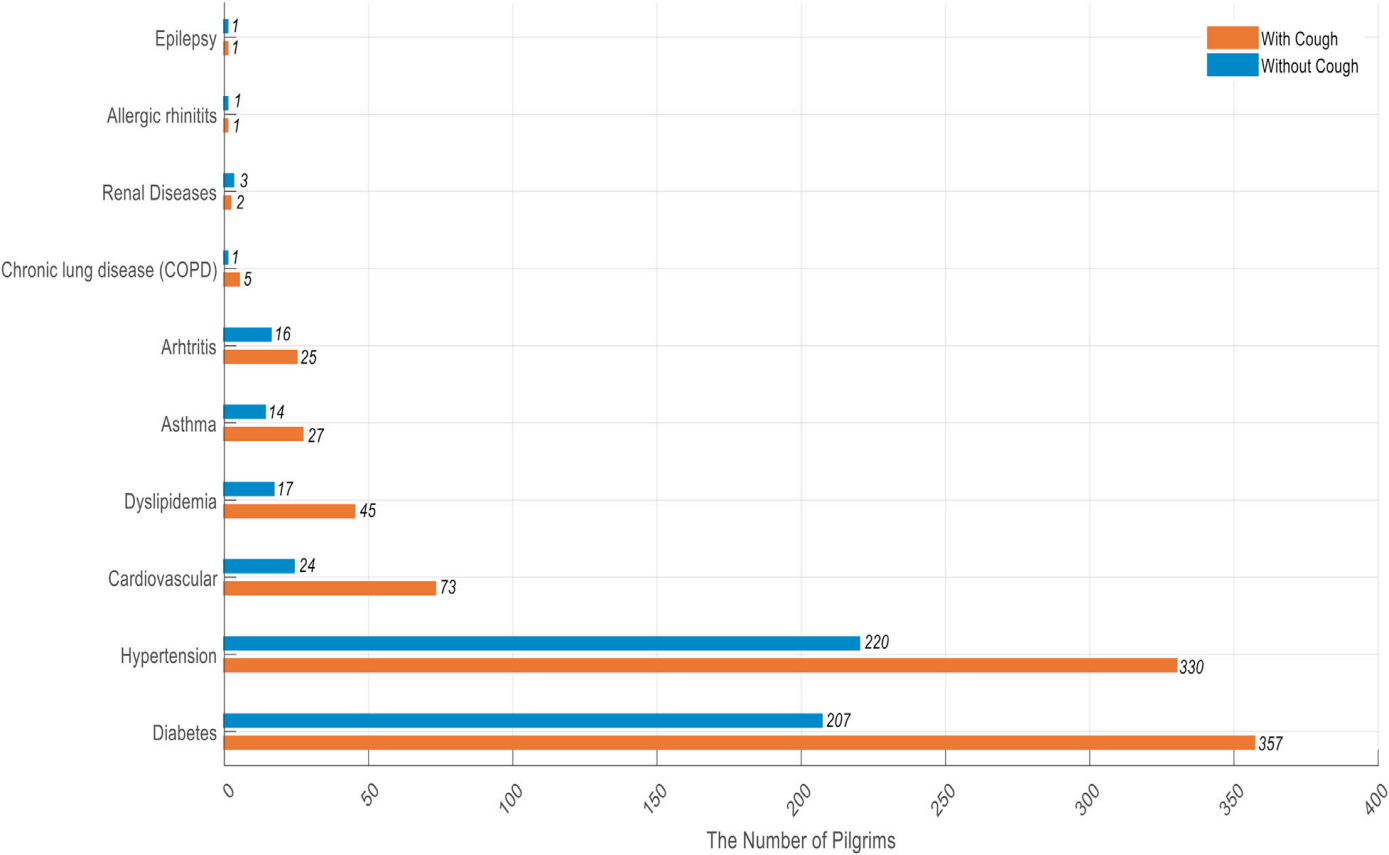
The number of Hajj pilgrims with chronic diseases based on the have or absence of cough.

Figure 1 presents an overview of chronic diseases among Hajj pilgrims, highlighting the differences between those with and without cough. Notably, diabetes mellitus was the most prevalent condition, with 357 pilgrims reporting coughs compared to 207 without cough. Similarly, hypertension was common, with 330 pilgrims experiencing cough, while 220 did not.

### Cough symptoms

Table 2 illustrates the distribution of associated symptoms among 1,176 participants with cough. Notably, 10.3% of individuals reported no associated symptoms, while 35.9% experienced one associated symptom. Additionally, 31.0% of participants reported two symptoms, and 22.7% presented with three or more symptoms. Among the associated symptoms, sore throat was the most prevalent, affecting 30.8% of participants, followed by a runny or stuffy nose in 21.7%. Other significant symptoms included sputum production (11.3%), hoarseness (10.6%), and fever (9.55%). Less common symptoms were shortness of breath (3.73%), wheezes (2.44%), and heartburn with regurgitation (1.59%).

**Table 2:** The distribution of associated symptoms with cough (N = 1176).

	N	% Total	% of other associated symptoms without cough
**The number of associated symptoms:**			
0	121	10.3	
1	423	35.9	40.1
2	365	31.0	34.5
≥3	267	22.7	25.3
**Symptoms**			
Sore throat	693	30.8	32.5
A runny or stuffy nose	488	21.7	22.9
Sputum	255	11.3	11.9
Hoarseness	239	10.6	11.2
Fever	215	9.55	10.1
Shortness of breath	84	3.73	3.9
Wheezing	55	2.44	2.6
Heartburn & regurgitation	36	1.59	1.7
Increased with physical activity	32	1.42	1.5
Vomiting	19	0.84	0.9
Increased with lying down	13	0.58	0.6
Coughing up blood	2	0.09	0.09

### Duration of cough over time

**Figure. 2:**
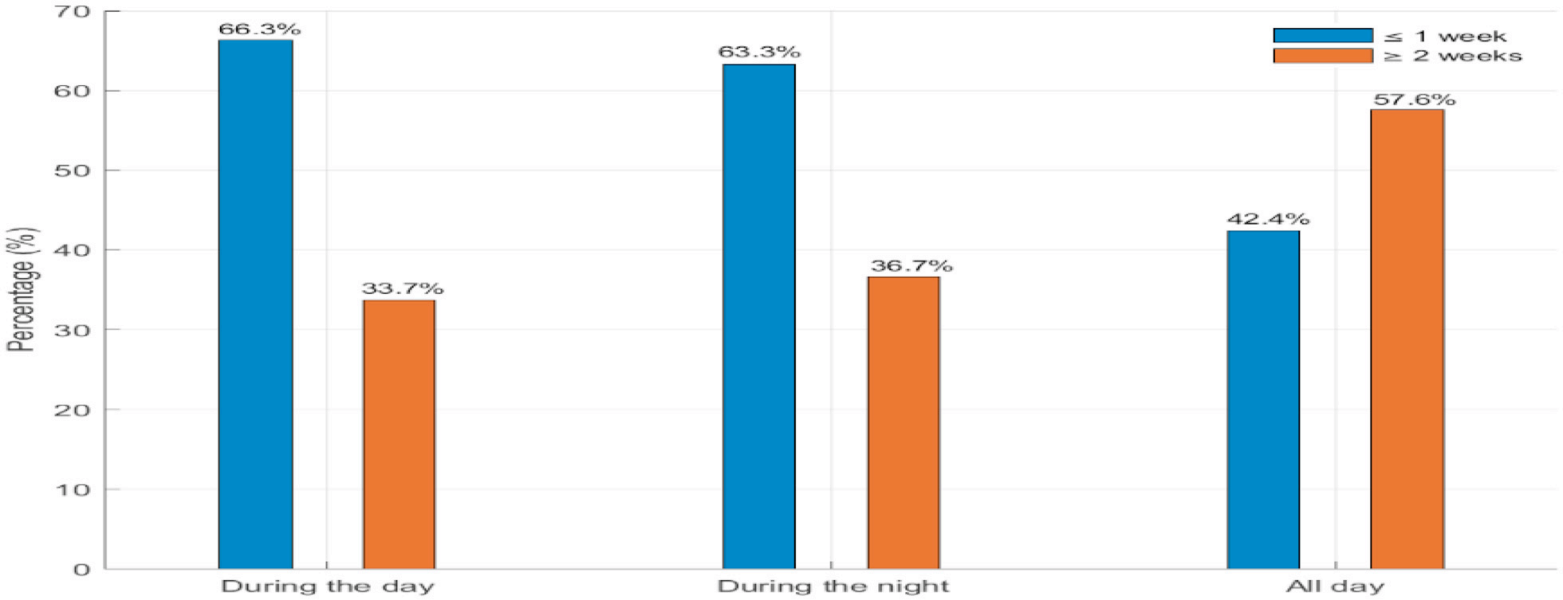
Distribution of cough patients based on cough duration and time of day.

Figure 2 illustrates the distribution of cough based on duration and day-night pattern. During the day, 66.3% of patients reported experiencing a cough lasting 1 week, while 33.7% indicated that their symptoms lasted for 2 weeks or longer. A similar trend was observed at night, with 63.9% of patients reporting a 1-week cough duration, compared to 36.7% who reported a longer duration. In the “All day” category, the figures shifted slightly, with 57.6% reporting a 1-week cough and 42.4% reporting a duration of 2 weeks or more.

### Factors influencing the prevalence of cough among Hajj pilgrims

The results from univariate logistic regression reveal that having a chronic disease significantly increases the likelihood of experiencing a cough, with an OR of 3.68 (95% CI 3.14-4.32; *P* < 0.0001) (Table 3). Nationality was also associated with prevalence, exhibiting an OR of 1.07 (95% CI 0.05-0.08; *P* < 0.0001). Furthermore, age was a contributing factor, with an OR of 1.004 (95% CI 0.003-0.006; *P* < 0.0001). In the multivariate logistic regression analysis, chronic disease, nationality, and age continued to be significant independent predictors of cough prevalence, with ORs of 1.31 (95% CI 0.23-0.32; *P* < 0.0001), 1.03 (95% CI 0.02-0.04; *P* < 0.0001), and 1.00 (95% CI 0.0012-0.004; *P* = 0.00024), respectively.

**Table 3:** Factors associated with the prevalence of cough.

Characteristic	Univariate analysis		Multivariate analysis	
	Unadjusted OR (95% CI)	*P*-value	Adjusted OR (95% CI)	*P*-value
**Age, (range 18 – 94), years**	**1.004 (0.003-0.006)**	***P* <0.0001**^a^	**1.00 (0.0012** – **0.004)**	**0.00024**^a^
≤29	0.52 (0.25-1.05)	0.069		
30-49	Reference			
50-64	1.51 (1.27-1.80) *P* <0.0001^a^			
≥65	1.46 (1.19-1.79) 0.0003 a			
**Sex**			**1.46 (–0.033 - 0.031)**	**0.961**
Male	Reference			
Female	1.05 (0.91-1.22)	0.5055		
**Education level**	**0.905 (–0.11 - –0.09)**	***P* <0.0001**^a^	**0.908(–0.12 - 0.08)**	***P* <0.0001**^a^
I preferred not to say	Reference			
University	3.74 (2.98-4.69)	*P* <0.0001^a^		
Read & write	5.86 (4.55-7.56)	*P* <0.0001^a^		
Illiterate	4.72 (3.47-6.41)	*P* <0.0001^a^		
Intermediate or Secondary	4.57 (3.61- 5.79)	*P* <0.0001^a^		
**Nationality by World Health Organization**	**1.07 (0.05-0.08)**	***P* <0.0001**^a^	**1.03 (0.02-0.04)**	***P* <0.0001**^a^
Eastern Mediterranean Region	0.46 (0.31-0.68)	0.0001^a^		
South-East Asia Region	0.15 (0.10-0.23)	*P* <0.0001^a^		
European Region	0.22 (0.14-0.34)	*P* <0.0001^a^		
African Region	0.27 (0.18-0.39)	*P* <0.0001^a^		
Western Pacific Region	Reference			
**No chronic disease** Reference				
**Chronic disease 3.68 (3.14-4.32) *P* <0.0001**^a^	**1.31(0.23- 0.32)**	***P* <0.0001**^a^		
Diabetes	3.22 (2.66-3.9)	*P* <0.0001^a^		
Hypertension	2.69 (2.22-3.25)	*P* <0.0001^a^		
Cardiovascular	4.72 (2.96-7.54)	*P* <0.0001^a^		
Dyslipidemias	4.02 (2.29-7.1)	*P* <0.0001^a^		
Asthma	2.89 (1.51-5.54)	0.0014^a^		
Arthritis	2.34 (1.24-4.4)	0.0085 a		
Chronic obstructive pulmonary disease	7.41 (0.86-63.5)	0.0676		
Renal diseases	0.98 (0.16-5.9)	0.986		
Allergic rhinitis	1.47 (0.092-23.6)	0.78		
Epilepsy	1.47 (0.092-23.6)	0.78		

CI, confidence interval; OR, odds ratio.

a Statistically significant (*P* <0.05).

## Discussion

This study evaluated the prevalence, duration, and associated symptoms of cough among Hajj pilgrims during the 2024 season by employing a standardized methodology to ensure both consistency and reliability. The findings revealed significant variations in cough prevalence based on age and nationality, while sex did not appear to influence these outcomes. Cough was reported in 40% of pilgrims, which is higher than the 7.5% reported in a previous study [19]. Specifically, 60.7% of pilgrims aged 50-64 and 27.9% of those aged 30-49 years reported cough symptoms. These results are consistent with the findings of a notably high prevalence of cough among individuals aged 45-64 years [12]. Similarly, Lai et al. [20] found that irrespective of sex, older age pilgrims exhibited more cough compared to their younger counterparts.

Moreover, the study's observation of an approximately equal male-to-female ratio in cough incidence suggests an absence of sex disparity in this context. This finding contrasts with that of Gautret et al. [12], who reported that females were more likely than males to experience Hajj-related cough. Additionally, Lai et al. [20] noted that a more significant proportion of females demonstrated hypersensitivity to environmental cough triggers. This discrepancy underscores the necessity for further investigation of sex-specific responses to environmental and health factors during pilgrimages. The study revealed that 32.8% of participants originated from the Eastern Mediterranean Region, where 41.7% reported cough symptoms. This observation is consistent with the findings of Abdo et al. [21], who noted that countries in the EMR are significantly affected by environmental stressors such as dust storms and elevated levels of particulate matter concentration. These environmental factors, in conjunction with the unique climatic, topographic, and socio-economic characteristics of the region, may contribute to the observed prevalence of cough among pilgrims from this area.

Chronic diseases also play a critical role in the emergence of cough symptoms among pilgrims. The results indicated that diabetes mellitus was the most prevalent condition, with 357 pilgrims reporting coughs compared to 207 without symptoms. Supporting this, Guilleminault et al. [22] noted a correlation between diabetes mellitus and the development of chronic cough. Additionally, Song et al*.* [23] stated that uncontrolled diabetes mellitus is a potential risk factor for cough. Hypertension also presented notable figures, with 330 pilgrims experiencing coughs, though this was lower than the prevalence associated with diabetes. This discrepancy may be attributed to the fact that hypertensive patients with ACE-inhibitor-induced cough exhibited significantly lower incidence, frequency, and severity of dry cough when treated with candesartan cilexetil compared to enalapril [24].

The majority of participants with coughs experienced shorter durations, with 66.3% reporting a cough lasting only 1 week. This suggests that the severity of cough may be lower in most cases. Such findings may be indicative of viral infections, as Witek et al. [25] found that symptom reporting typically commenced within 24 hours of cold onset for most subjects. Therefore, immunization is vital during large MGs, such as Hajj, to prevent or mitigate the severity of respiratory illnesses that contribute to cough occurrence [26]. Pre-Hajj screening and health education are also crucial as effective preventive measures[27]. In their study, a sore throat was identified as an early indicator of illness, often accompanied by various symptoms, including nasal congestion, a runny nose, and headache.

### Strengths and limitations

The study acknowledges several limitations. Although the sample encompassed participants from 40 countries, the majority were from 12 nations, which may not fully capture the diversity of experiences among all Hajj pilgrims. This limitation is due to the time constraints and the study’s setting in Jeddah, where a significant proportion of pilgrims depart from Madinah, making it challenging to definitively ascertain the direction of potential selection bias. Recall bias is also likely in this study due to the retrospective nature of data collection. The questionnaire did not include smoking status because of frequent inaccuracies in responses, attributed to the diverse types of smoking that participants either did not recognize as smoking or chose not to disclose during the pilot phase. In addition, we did not collect data on vaccinations against respiratory infections or on the use of facemasks. Despite the use of translators, cultural nuances in expressing mental health symptoms may have led to misclassification or underdiagnosis, particularly among non-Arabic or non-English-speaking pilgrims.

## Conclusion

The present study examined the prevalence of cough and associated symptoms among pilgrims during the large religious MGs of the 2024 Hajj season, yielding significant findings that contribute to our understanding of respiratory health in this unique population. The analysis revealed that nearly two in five pilgrims reported cough symptoms, with the highest prevalence found among the 50–64 age group, where 60.7% experienced this symptom. Furthermore, a substantial association was identified between cough and chronic diseases, as 53.3% of those affected had pre-existing health conditions, predominantly diabetes and hypertension. The symptomatology associated with cough was also noteworthy; while 10.3% of participants reported cough in isolation, sore throat emerged as the most common additional symptom, affecting 30.8% of those with cough. Logistic regression analysis provided further insight, establishing chronic diseases, age, and nationality as significant predictors of cough among the participants. The results of this study indicate that respiratory health among pilgrims during the Hajj season is significantly influenced by age and the presence of chronic diseases. This work contributes to existing knowledge by providing empirical data on the prevalence of cough and its associated factors in a high-density pilgrimage setting. More broadly, future research is needed to determine the long-term health outcomes for pilgrims post-MGs and to explore the impact of environmental factors, such as air quality and crowd density, on respiratory health during large mass gathering events.

## Declaration of competing interest

The authors have no competing interests to declare.
